# Exploring rounD Dataset for Domain Generalization in Autonomous Vehicle Trajectory Prediction

**DOI:** 10.3390/s24237538

**Published:** 2024-11-26

**Authors:** Zikai Zhang

**Affiliations:** Department of Computer Science, Durham University, Stockton Rd, Durham DH1 3LE, UK; zikai.zhang@durham.ac.uk

**Keywords:** machine learning, domain generalization, driving behavior, motion forecasting, trajectory prediction

## Abstract

This paper analyzes the rounD dataset to advance motion forecasting algorithms for autonomous vehicles navigating complex roundabout environments. We develop a trajectory prediction framework inspired by Gated Recurrent Unit (GRU) networks and graph-based modules to effectively model vehicle interactions. Our primary objective is to evaluate the generalizability of the proposed model across diverse training and testing datasets. Through extensive experiments, we investigate how varying data distributions—such as different road configurations and recording times—impact the model’s prediction accuracy and robustness. This study provides key insights into the challenges of domain generalization in autonomous vehicle trajectory prediction.

## 1. Introduction

The autonomous vehicle (AV) industry is poised to transform mobility as we know it today, and market projections are expected to be felt worldwide. Motion forecasting is one of the key technologies enabling this transformation, as it gives AVs the ability to predict where other entities like vehicles, pedestrians and road users will be in the future [1,2]. As it provides this information to the decision-making algorithms, which in turn help actions like lane changes and collision avoidance, AVs are safe and operational with respect to them [3,4].

However, the field struggles a lot. The domain gap between the specialized clips used to train a model and what it perceives as real-world sensory input during driving is one of many major issues. With the motion forecasting landscape for autonomous vehicles being quickly revolutionized, it is becoming increasingly critical to deal with these domain discrepancies. Here, we introduce transfer learning and domain adaptation, which are two prominent methods aimed at addressing domain discrepancies in machine learning. Transfer learning involves taking data from one domain and leveraging them to enhance the performance of a model in another domain. Similarly, domain adaptation is a technique designed to mitigate source-sample training bias and test-time overfitting by transferring knowledge across domains, particularly when bridging data distributions from known sources to unfamiliar targets [5]. However, both of these methods pose challenges in the context of autonomous vehicle (AV) trajectory prediction. They require additional data from new scenarios to update the model, which can be difficult to obtain. In the case of AV trajectory prediction, the real-world driving environment is constantly changing and is highly dynamic, making it challenging to collect comprehensive datasets that cover all possible scenarios. Therefore, domain generalization emerges as a promising solution. Unlike transfer learning and domain adaptation, domain generalization does not rely on new data from unseen scenarios. Instead, it involves training models across multiple source domains to achieve performance that can generalize well when deployed in an unseen environment. This approach is particularly crucial for AVs operating in complex and varied environments, as it enables them to adapt to new situations without requiring extensive additional data. Despite its potential, domain generalization also presents significant challenges, particularly in consistently generalizing across two completely different worlds. Validating the effectiveness of these approaches in real-world driving environments, which are characterized by a high degree of scenario variance, remains extremely difficult. This underscores the need for continued research and development in this area, as autonomous vehicles must not only navigate but also become some of the safest entities on the road [6].

Current leading forecasting methodologies sometimes fail to be effectively implemented in real-world situations because they rely too heavily on datasets that oversimplify reality [7,8]. Problems like incorrect detection/tracking or poor quality sensor information lead to sizable discrepancies in ground-truth data, which can also hamper the effectiveness of motion forecasting models [9,10]. Furthermore, many models do not use relevant road map and environmental context information from perception modules to make precise predictions in non-stationary or highly complex environments [11]. Perception input does not provide perfect quality and fidelity depending on object distance from the ego vehicle, which is another challenge that existing forecasting benchmarks tend to ignore [7]. As a result, end-to-end data analysis should become increasingly important, helping us identify where errors occur within the process and what improvements are needed to develop fully functional autoregressive models for real-world driving [7,8]. Moreover, a global evaluation scenario is required to unify the experimental design and facilitate comparisons between traditional methods and end-to-end forecasting [12]. Finally, it is important to understand the impact of different data distributions on models. Factors such as urban versus rural environments, or variations in daytime versus nighttime conditions and weather, can significantly affect model effectiveness [13]. For example, models trained with predominantly urban data may be very inaccurate in the countryside, and clear weather models fail to classify foggy or rainy photos correctly [14]. Therefore, to ensure models are not conducting capturing unfairly, this bias and can generalize well enough for broader life-critical cases. Researchers desperately need more robust motion forecasting models that incorporate the effects of data distribution as part of model design [7], especially in the case of autonomous vehicles (AVs), which are hoped to function under a variety of environment and environmental conditions. Therefore, to advance the field, it is not merely beneficial but absolutely necessary to scrutinize how different data distributions influence model performance [15].

This paper will use a backbone model including GRU and q graph-based method to deal with the trajectory prediction task. In particular, a distribution analysis of several features is performed by dividing the rounD dataset into several groups that uniformly cover all mentioned traffic scenarios. Contrary to previous works, a thorough cross-recording comparison is contributed within the rounD dataset. With this more nuanced approach, the specific challenges or opportunities related to each traffic condition can be targeted, thereby improving the model’s capacity for accurate predictions of future trajectories. This analysis of driving data and its influence on prediction models provides deep insights for the field of motion prediction in autonomous driving.

## 2. Related Work

### 2.1. Trajectory Prediction

Trajectory prediction is fundamental for self-driving vehicles to navigate complex, dynamic scenes safely and efficiently. Deep learning architectures like Generative Adversarial Networks (GANs) and Variational Autoencoders (VAEs) are commonly used for motion forecasting. For example, Social GANs produce socially aware trajectories by modeling fine-grained interactions among road agents, effectively matching human drivers’ actions [16]. VAEs capture intrinsic motion uncertainty by predicting probability distributions over possible future trajectories, enhancing self-driving capabilities in complex contexts [17]. State-of-the-art approaches like Target-driveN Trajectory Prediction (TNT) and DenseTNT emphasize temporal features to maintain consistent predictions over time [18,19]. LaneRCNN fuses lane information with road semantics to constrain predictions within reasonable spaces [20]. MultiPath and MultiPath++ predict multiple future trajectory options to handle uncertainties [21,22]. Recently, graph-based networks have become key techniques for trajectory prediction due to their ability to capture complex interactions. GRIP (Graph-based Interaction-aware Trajectory Prediction) introduced graph structures to model agent interactions [23], leading to rapid developments in the field. Successors like GRIP++ [24] and GISNet [25] further improved graph-based models by considering both spatial and temporal aspects. Additionally, to enhance the integration of multi-modal data for trajectory prediction, [26] recently enabled the accurate alignment of image data with point cloud data, providing a richer context for understanding the surrounding environment. By leveraging the coarse-to-fine correspondences extracted by CoFiI2P, it is convenient to fuse visual information with spatial data, leading to more informed and accurate trajectory predictions. In our study, the structure of GRIP++ is used as the creative inspiration in this study due to its effectiveness in modeling complex spatiotemporal interactions using graph representations. By modifying the GRU modules and incorporating graph-based feature representations instead of direct coordinate inputs, a model that is sufficiently robust and representative is aimed to be used.

### 2.2. Driving Behaviors

Understanding how vehicles operate, especially in the context of autonomous driving, requires a deep dive into driving behavior. Various methods have been used to explore this complex field. For instance, scenario-adaptive techniques help us see how driving behavior changes in different contexts, offering valuable insights into how drivers react to various environments [27]. Researchers also look at the social aspects of driving, examining how vehicles interact with each other and with pedestrians. This helps in understanding the unwritten rules that drivers follow on the road [16]. Following these papers, a sophisticated analysis is conducted in multiple ways (e.g., time and agent interactions) to explore the factors related to driving behaviors. By using these diverse methods, we can better understand the intricacies of driving behavior, which is crucial for developing reliable and safe autonomous driving systems. Key factors influencing the analysis of driving behavior include the context in which driving occurs, the inherent unpredictability of human driving habits, and the dynamic interactions between vehicles, pedestrians, and the broader environment. These factors play a pivotal role in shaping the driving behavior observed in real-world scenarios [28].

### 2.3. rounD Dataset

The rounD dataset comprises video data obtained from a drone flying over roundabouts, which offers several advantages over onboard camera footage. Drones provide a broad, bird-eye view, capturing the entire roundabout and surrounding traffic with minimal occlusion, allowing for a comprehensive analysis of traffic flow and vehicle interactions across multiple lanes. This aerial perspective reduces the limitations of onboard cameras, which are often obstructed by the vehicle itself. Drones also offer flexibility in repositioning and adjusting altitude to capture different angles, providing a clearer, more stable view of traffic patterns and behaviors. This makes drone footage particularly valuable for analyzing trajectory prediction tasks that involve interactions among multiple agents in complex environments, while onboard cameras are better suited for real-time autonomous vehicle navigation and decision making. Using the roundD dataset, the Scout study proposed a social-consistency-aware graph attention network for vehicle and Vulnerable Road User (VRU) motion forecasting [29]. Additionally, several other studies have leveraged roundD to develop deep learning-based architectures for trajectory prediction and correction, highlighting its significance in achieving reliable forecasting for safe vehicle navigation. Furthermore, the multi-agent GRIT method for goal recognition—an approach not widely adopted in this field but enabling fast and interpretable predictions—has been applied using roundD data to enhance autonomous driving scenarios [30]. In this work, we focus on assessing the generalizability of our proposed trajectory prediction model across diverse training and testing data using the rounD dataset.

### 2.4. Domain Generalization

In model training tasks, data distribution is important—especially in domain generalization. This idea is crucial for making sure that our models are still able to be flexible and generalizable across new environments they have never seen. Ref. [31] provided a valuable perspective on domain generalization, highlighting that it is one of the main challenges in ensuring that models perform effectively across different situations. It is a major pain point in applications like autonomous driving or industrial automation. To improve on this, the study presented in [32] introduces a fundamentally new approach to trajectory prediction for autonomous vehicles. Their solution is a Graph Neural Network (GNN)-based variant that incorporates domain generalization, the unsolvable problem of universal alignment and adaptation to unseen driving scenarios. This work will adopt GNN-based techniques to deal with the feature representation part in the rounD dataset and launch a sophisticated domain generalization analysis between each driving scenario. This work is expected to lay the foundation for future research on the rounD dataset and emphasize the importance of domain generalization for the development of reliable autonomous systems [33].

## 3. Problem Formulation

We first formulate the trajectory prediction problem, which involves predicting the future locations of every object in a scene using their past trajectories. To be more specific, our model takes the trajectory histories of all observed objects over $T_{h}$ time steps as its inputs, denoted as I:

(1) $$I=[p_{1},p_{2},\ldots ,p_{T_{h}}]$$

where

(2) $$p_{T_{h}}=\left[x_{T_{h}}^{1},y_{T_{h}}^{1},x_{T_{h}}^{2},y_{T_{h}}^{2},\ldots ,x_{T_{h}}^{N},y_{T_{h}}^{N}\right]$$

This equation is a coordinates combination of all the observed agents at time $T_{h}$, and *N* is the total number of agents. Both world coordinates and relative measurements are utilized, such as agent-targeted coordinates. Our objective is to predict the complete trajectories of observed agents over the next $T_{f}$ steps, which will be represented as the output *O* of our model:

(3) $$O=[p_{t+1},p_{t+2},\ldots ,p_{t+T_{f}}]$$

Let I denote a nonempty input space and O the label space. A domain is defined as $S={\left\{\left(I_{i},O_{i}\right)\right\}}_{i=1}^{n}∼P_{IO}$, where $I\in I\subset R^{d}$, $O\in O\subset R^{d_{l}}$ denotes the label, and $P_{IO}$ represents the joint distribution of the input sample and output label. *I* and *O* denote the corresponding random variables. In domain generalization, *M* training (source) domains are given as $D_{train}=\left\{D^{i}\;\;i=1,\cdots ,M\right\}$, where $D^{i}={\left\{\left(I_{j}^{i},O_{j}^{i}\right)\right\}}_{j=1}^{n_{i}}$ denotes the $i^{th}$ domain and $I_{j}={\left(I_{t}^{j}\right)}_{t=1}^{T}={\left[\xi_{t}^{A,j}\right]}_{t=1}^{T}$ denotes a multivariate time series with $I_{t}^{i}\in R^{H\times 1}$. ξ is used to represent historical trajectories for a given vehicle *A* in the $i_{th}$ domain (map). In this work, the objective is to evaluate the developed predictor *f* to see if it can maintain its effectiveness across each unseen target domain $i^{\prime }$. (i.e., $i^{\prime }\notin D_{i}$, where $P_{IO}^{i^{\prime }}\neq P_{IO}^{i}$, ∀i∈{1,⋯,M}).

## 4. Overview of the rounD Dataset

For the roundD dataset, all the recordings are captured under optimal weather conditions, ensuring clear skies, ample lighting, and minimal wind, which enhanced the overall quality of the footage. This guaranteed greater image clarity and stability, simplifying subsequent processing tasks. All footage was recorded using a DJI Phantom 4 Pro drone, which boasts a camera capable of 4K resolution (4096 × 2160 pixels). The videos are shot at the highest bitrate, capturing 25 frames per second. It successfully extracted information on 13,746 road users, i.e., cars (11,530), followed by trucks and vans. Other vehicles like trailers and buses were less common. Vulnerable Road Users (VRUs), such as pedestrians, bicyclists, and motorcyclists, are rarer, likely because the roundabouts were situated away from urban centers or shopping districts. The data for each road user includes details like position, heading, speed, and acceleration in both *x* and *y* axes of the static UTM coordinate system, as well as in the longitudinal and lateral directions of each participant’s movement [34].

**Figure 1:**
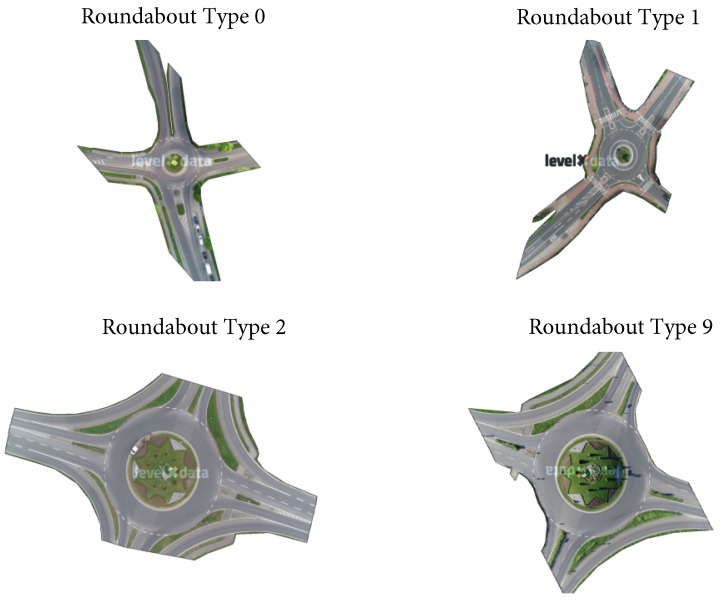
All four roundabout types in the rounD dataset. Here, roundabout type 0 is denoted as the background image from recording file 0 and so are roundabout types 1, 2 and 9.

In Figure 1, four different roundabout types are discovered from a total of 23 recordings. Among these, roundabout type 0 has a single lane for all entries and exits, and a circular island in the center. Although roundabout type 1 shares the same shape with Recording_00, the former is more complex with spiraled traffic lanes to guide vehicles into the correct lane before entering the roundabout. Moreover, the orientations of the entries and exits are different. The remaining two roundabout types (2 and 9) are topologically similar, differing only in their rotations and scales.

Here, Table 1 contains metadata for each recording file, where recording conditions and parameters are provided, for example, latitude and longitude of the recording location, UTM coordinate origin, and conversion factor from pixels to meters. Additionally, Table 2 provides detailed information for each tracked object, including position coordinates, heading angle, width, length, velocities and accelerations in different directions, start and end frames, total frames, and object class, etc.

## 5. Dataset Analysis

### 5.1. Analysis on Roundabout Type

To analyze the dataset, an understanding of the high-level data used is first required. For the four selected recordings, the following description demonstrates their corresponding map types (see Figure 1) and the corresponding amount of data in each recording.

In Table 3, there is only one file (Recording_00 and Recording_01) in roundabout types 0 and 1. Roundabout type 3 encompasses recordings numbered 2 through 8, accumulating to a total data amount of 1,732,504. Lastly, roundabout type 9 includes recordings numbered 9 to 23, summing up to a total data amount of 3,303,282.

### 5.2. Analysis on Agent Class Distribution

It is important to recognize the distribution of different agent classes within a given set of input data and why having this knowledge can facilitate the task at hand. What this allows for is a way to figure out who road users are and how they balance or impair traffic. It also contains some examples, like cars, trucks, and pedestrians. This distribution allows us to be aware of possible biases in the data and attempt to correct for them, so our models are not overly biased towards any one class. This is particularly challenging in motion forecasting fields for autonomous driving as predicting various behaviors of different agents is crucial to ensure reliability and safety. Improving the robustness and generalizability of our model across diverse real-world scenarios requires that all agent classes are represented in a balanced way.

**Figure 2:**
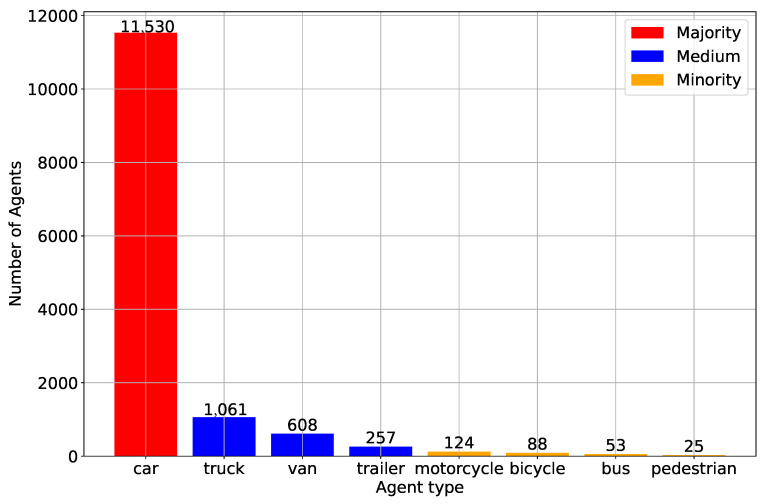
Class distribution of agents, categorized into ‘Majority’ (car), ‘Medium’ (truck, van, trailer), and ‘Minority’ (motorcycle, bicycle, bus, pedestrian).

In Figure 2, the distribution of various classes of agents is presented. The vertical axis represents the agent count, while the horizontal axis enumerates the different agent classes: car, truck, van, trailer, motorcycle, bicycle, bus, and pedestrian. Specifically, “car” is labeled as the ‘Majority’ class (red), and truck, van, trailer as the ‘Medium’ class (blue), which are smaller than 10% but higher than 1% of the majority. The ‘Minority’ class stands for the remaining four classes, i.e., motorcycle, bicycle, bus and pedestrian, which are less than 1% of the majority. Due to the limited number of observed samples and the significant differences in trajectories between motor vehicles, non-motor vehicles, and pedestrians, the decision has been made to exclude these classes from the analysis. As a result, without loss of generality, the focus will be on the interactions and influences between automobiles, rather than VRUs, to ensure the accuracy of predicted trajectories.

### 5.3. Analysis on Recording Time Distribution

The time of recording can significantly influence driving behavior due to factors such as traffic density and driver urgency. By analyzing the distribution of recordings across different times of the day, the aim is to understand how temporal variations are represented in the dataset. This knowledge helps us to evaluate whether our model can adapt to different driving conditions encountered at various times. Ensuring that our models perform well under diverse temporal conditions enhances their applicability in real-world scenarios and improves their overall robustness.

**Figure 3:**
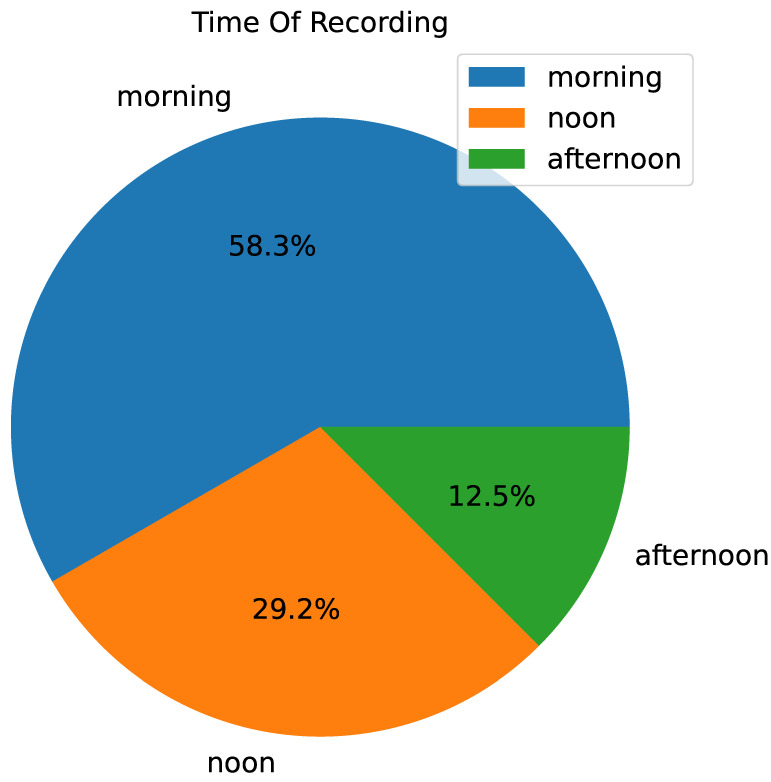
Time distribution of recordings, divided into morning, noon, and afternoon categories, highlighting variations in driving behaviors.

**Figure 4:**
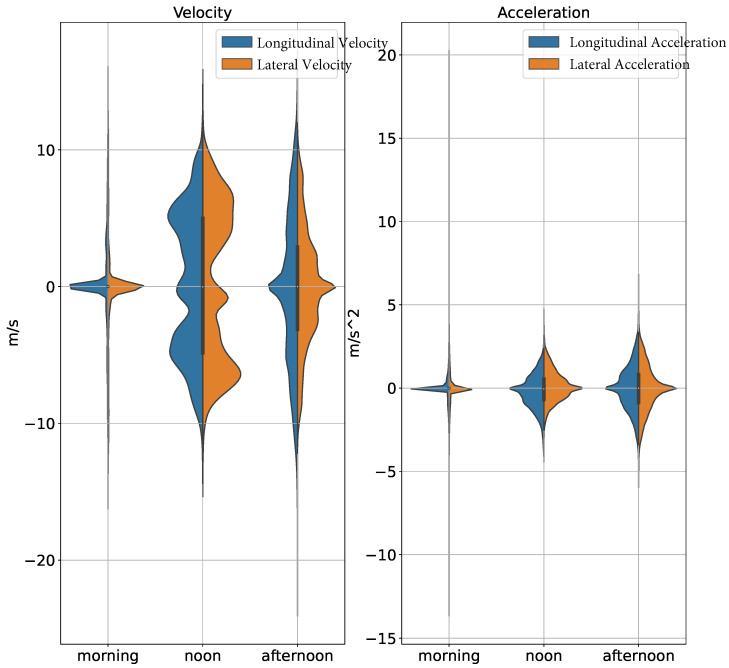
Comparison of morning and afternoon driving behaviors: wider velocity and acceleration ranges are observed in the morning, indicating more aggressive, stop-and-go driving. Noon and afternoon driving shows higher velocities but more consistent, relaxed acceleration patterns.

In Figure 3, the distribution of recordings based on the time of day is illustrated. The pie chart divides the data into three primary categories: morning, noon, and afternoon. This means that most recordings in the rounD dataset start in the morning, with approximately 30% at noon and only 10% in the afternoon. This type of distribution offers some hints about the effects that recording times can have on driving behaviors. This figure also shows different trajectory patterns in morning rush hours, possibly because drivers would drive more aggressively or think about their works, in comparison to midday or afternoon, as it can be seen from Figure 4. The afternoon, and especially weekends, might be a time when drivers are less aggressive due to family outings [35]. Knowing these day-dependent differences is essential when creating robust prediction models that can adjust to the driving behavior at different times of a single drive [36].

### 5.4. The Effect of Recording Time on Vehicle State

This section continues exploring how the timestamp alters vehicle states. The idea is to find patterns when they happen at different times of the day, and then to predict how these will affect motion forecasting. Understanding these effects is essential for modeling time-varying driving behavior.

In Figure 4, it can be observed that the variation in acceleration and speed in the morning is much greater than in the noon and afternoon, suggesting that driving in the morning tends to do quick starts and stops. Perhaps due to the need for mobility, drivers tend to accelerate more and make frequent adjustments to their speed to meet commuting needs. On the other hand, velocities in the noon and afternoon suggest that the road conditions are good to accommodate different driving styles, with most driving tending to be at a constant speed. Moreover, the degree of acceleration variation at noon is smaller than in the afternoon, which means that the driving behavior at noon could be more relaxed, with less variability in acceleration, perhaps due to a lower pressure of having to reach a destination quickly. Meanwhile, the afternoon plot appears to have a wider distribution at higher velocities, possibly indicating faster driving speeds. However, the range for acceleration is much less wider than that in the morning, meaning that drivers choose to travel at a relatively constant speed rather than aggressively.

### 5.5. The Effect of Driving Behaviors on Vehicle States

Understanding the impact of driving behaviors on vehicle states is crucial for developing accurate motion forecasting models. By analyzing how different driving behaviors, such as acceleration, deceleration, and constant speed, influence vehicle states like velocity and acceleration, valuable insights into the dynamics of driving patterns can be gained. This analysis helps us to identify the variations in driving behaviors across different times of the day and under various traffic conditions. By incorporating these behavioral patterns into our model, the accuracy of vehicle movement predictions can be improved. This step is essential for enhancing the robustness and reliability of our model, ensuring they can effectively handle diverse driving scenarios and provide precise predictions in real-world applications.

**Figure 5:**
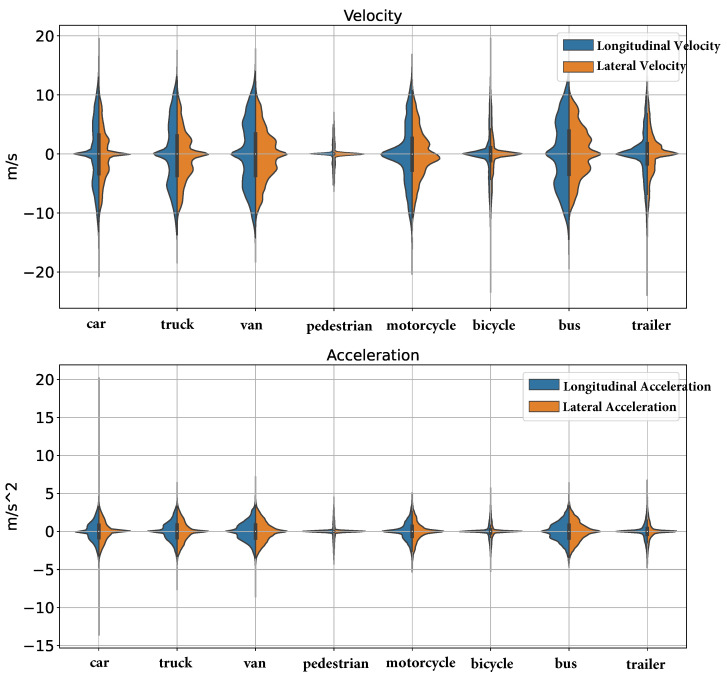
Velocity and acceleration comparison of road agents and VRUs: road agents (e.g., car, truck, van, motorcycle, bus) exhibit a much wider range than VRUs. Both velocity and acceleration show similar trends, indicating their comparable impact in motion forecasting.

According to Figure 5, in the top plot (Velocity), the velocity ranges of road agents are much larger than VRUs. For example, the variations in speed and acceleration of a trailer, bicycle, and pedestrian are much smaller compared to a car. Additionally, the acceleration ranges of road agents are also larger than VRUs. Therefore, it can be concluded that velocity has a similar impact as acceleration on the input of the rounD dataset.

### 5.6. Analysis of Feature Correlations

Analyzing the correlations between different features in our dataset provides valuable insights into the relationships among various attributes. This step helps in identifying redundant or highly correlated features, which can be used for feature selection or dimensionality reduction. By understanding these correlations, the efficiency and performance of the model can be improved, ensuring they are trained on the most informative features, thereby enhancing prediction accuracy and simplifying model complexity.

**Figure 6:**
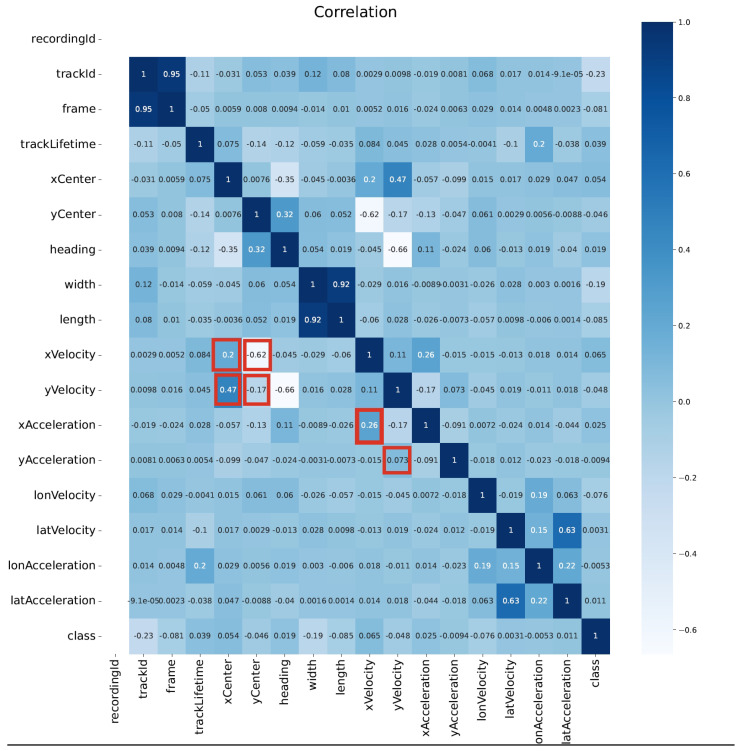
Correlation matrix heatmap: darker colors and lighter colors indicate larger positive and smaller negative correlations between indicators. Notable relationships include connections between xCenter, yCenter, xVelocity, and yVelocity, as well as between velocity and acceleration (noted in red rectangles). These findings suggest potential redundancy and highlight areas for feature extraction or dimensional reduction.

In Figure 6, the correlation matrix shows the correlation between each indicator and checks if there are high correlations between them. Warmer colors (like white and light blue) represent smaller negative values, while cooler colors (like dark blues and blue) represent larger positive values. A deep insight into the relationship between those attributes can be gained from this. Several key connections between the input of the model were identified. Both xCenter and yCenter are connected to xVelocity and yVelocity with correlation coefficients of 0.2, 0.47, and −0.62, −0.17, respectively. Additionally, xVelocity and yVelocity are correlated with the corresponding acceleration values, with coefficients of 0.26 and 0.073. Therefore, there is a conclusion that the pair of center points has deep connections with velocity.

### 5.7. Analysis Across Different Scenarios/Maps

To comprehensively evaluate our models, it is essential to test them across different scenarios and maps. This analysis aims to compare the performance of our models in varied environments, identifying any specific challenges or opportunities presented by different traffic conditions. By doing so, the generalizability and robustness of our models can be assessed, ensuring they can effectively handle diverse real-world scenarios. This enhances the practicality and reliability of our model in real-world applications.

In this part, two different scenarios (roundabout type 1 and 2) are selected and compared with each other in various aspects, including class distribution, sample trajectory, and average speed, which is important for analyzing the challenges in motion forecasting.

**Figure 7:**
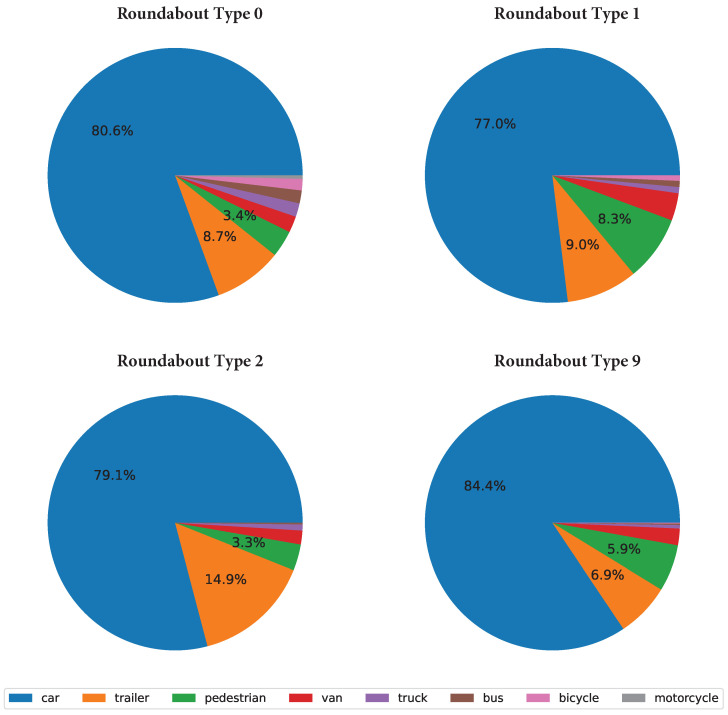
Pie chart analysis: cars dominate across all scenarios (over 75%) according to all recordings. Missing classes (some classes cannot be observed in a certain roundabout type) and VRUs, like trailers, bicycles, and pedestrians, are present in low percentages.

In Figure 7, the dominant agent in all scenarios is the car (accounting for more than 75%), and roundabout type 9 tends to have fewer classes of road agents than the other recordings do. To be more specific, some kinds of vehicles and VRUs are missing in roundabout type 9, for example, motorcycles. Therefore, without loss of generality, the nuance in both cases in terms of motion forecasting can be ignored, as the focus is on the movement of agents rather than the class.

(4) $$behavior=\left\{\begin{array}{cc} \mathrm{acceleration} & \mathrm{if}\;f(K_{i})>\theta \\ \mathrm{constant} & \mathrm{if}\;-\theta \leq f(K_{i})\leq \theta \\ \mathrm{deceleration} & \mathrm{if}\;f(K_{i})<-\theta \end{array}\right.$$

**Figure 8:**
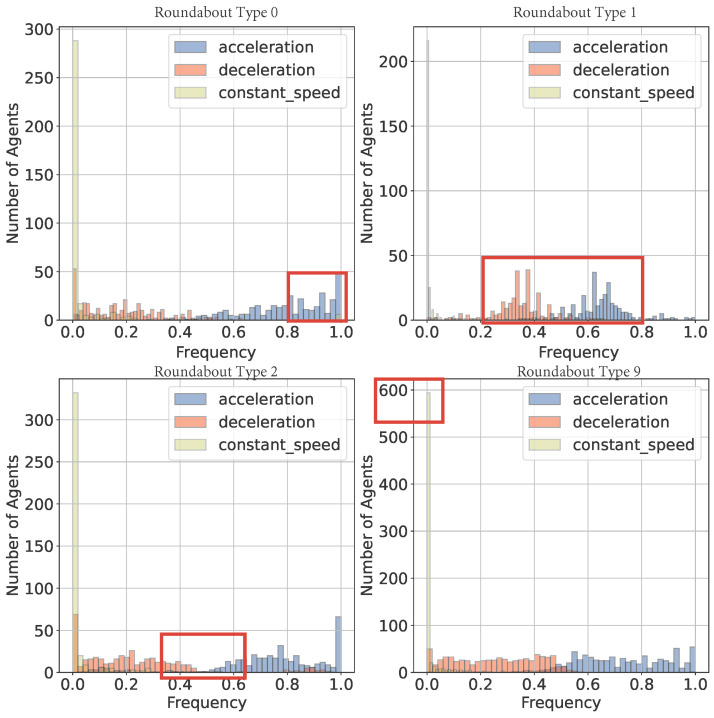
Acceleration and deceleration frequencies vary across recordings in the roundabout scenario, with deceleration generally being more common. Different trends are observed (marked in red), such as roundabout type 2’s unique pattern, roundabout type 1’s emphasis on specific acceleration ranges, and roundabout type 9’s tendency for constant speed.

Equation (4) is used to determine the class of behavior, where $K_{i}$ stands for the current state of agent *K* on frame *i*, *f* represents the evaluate function of driving behavior, which, for this case, is exactly the acceleration value on both x and y directions. Here, θ=0.05 is set as a hyperparameter because the accelerations of approximately 80% of agents are lower than 0.05, as shown in Figure 8.

In Figure 8, it can be found that in the roundabout scenario, few road agents will remain at a constant velocity during the trip, while the distributions of acceleration and deceleration are shown to be different. The frequency of deceleration is much larger than that of acceleration on all conditions, and one significant difference between the four roundabout types is the trend of deceleration and acceleration, especially in roundabout type 2. While those in roundabout type 1 focus mainly on the ranges [0.2, 0.4] and [0.6, 0.8], the other three recordings seem to be evenly distributed. However, in roundabout type 9, more agents tend to move at a constant speed during their trips, and agents in roundabout type 0 are more likely to accelerate rather than decelerate. The main reason for these differences is considered to be different road conditions, such as the number of crossroads, the number of intersections into the main road, and the number of agents at that time, which is also an important factor that has a great impact on motion forecasting.

## 6. Model Architecture

### 6.1. Network Overview

**Figure 9:**
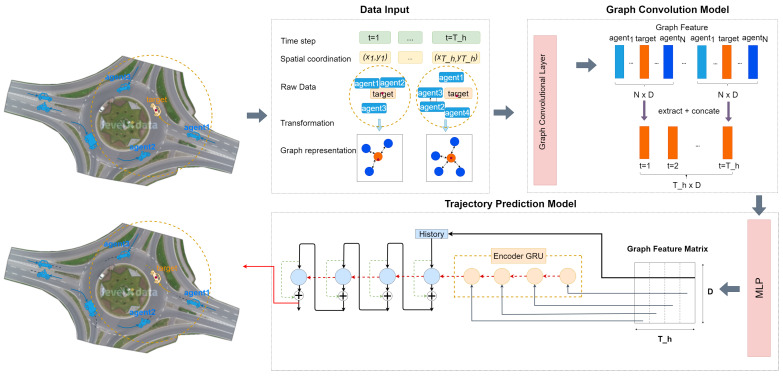
Overall architecture of the backbone trajectory prediction model used in our paper. Three main modules are included: data input, graph convolution model, and trajectory prediction model. Details of each model can be found in Figure 10 and Figure 11.

Figure 9 shows the backbone model we used for trajectory prediction. The trajectories of all road agents are illustrated, with the target being highlighted within the traffic graphs. The circle indicates that all agents inside, including the target, are represented as a fixed graph. This graph input is then transformed into several neighboring eigenvectors (each shade of blue representing an eigenvector index) and target eigenvectors of the original traffic graph. Subsequently, the target eigenvector (2-dimension) is extracted to create a new feature map across all $T_{h}$ frames. Using an encoder and decoder, both based on GRU architectures, the final trajectory can be predicted. Here, *D* denotes the batch size.

**Figure 10:**
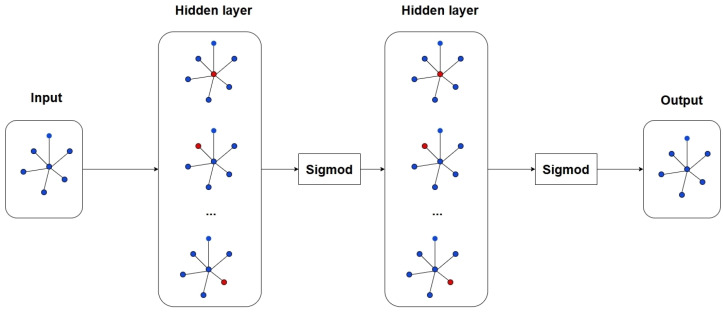
Details of the graph convolution model. The model is based on a Graph Convolution Network (GCN) structure. The input layer represents the initial node features (source node, neighboring node), which are passed through multiple hidden layers, each applying a graph convolution operation followed by a sigmoid activation function. The hidden layers capture the relationships between nodes by iteratively aggregating neighboring node information. The output layer provides the final representation of the node features after transformation through the network.

**Figure 11:**
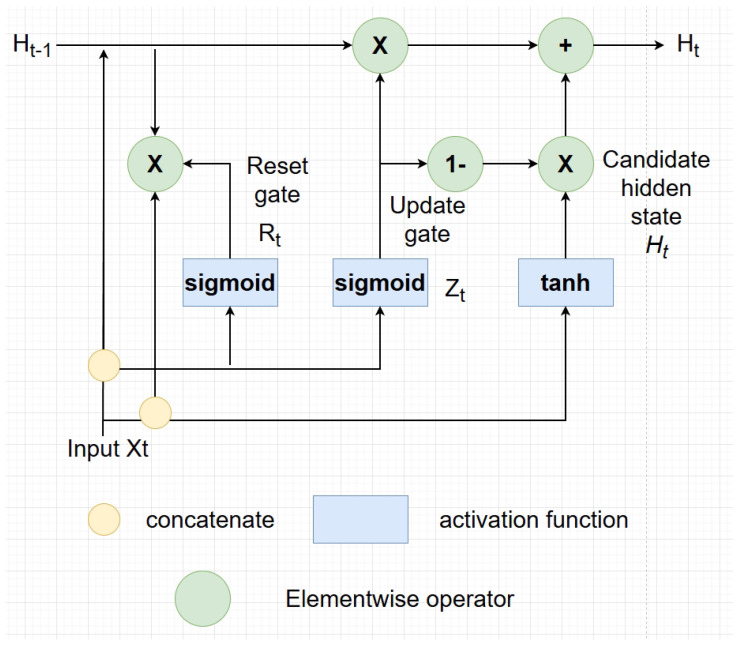
Details of the trajectory prediction model. A Gated Recurrent Unit (GRU) is utilized to capture the hidden information within the sequential input trajectories. The GRU architecture consists of reset and update gates, which control the flow of information. The reset gate $R_{t}$ determines how much of the previous hidden state $H_{t-1}$ to forget, while the update gate $Z_{t}$ balances between the previous hidden state and the candidate hidden state $H_{t}$, calculated using a tanh activation function. By combining the previous state and new information, the GRU captures dependencies without the need for separate cell states, as in LSTMs. In our paper, two Gated Recurrent Units are used.

### 6.2. Graph Representation

Each timestamp is represented with *n* observed road agents using a graph, with the local coordinates of the road agents representing the set of vertices $V=\{v_{1},v_{2},\ldots ,v_{n}\}$ and a set of undirected, weighted edges $E=\{e_{1},e_{2},\ldots ,e_{n}\}$. All the road agents in this timestamp are considered to be connected with the target agent through bi-directional edges. (For target agent *a* and for each other agent node *i*, $e_{a,i}$ and $e_{i,a}$ are obtained.) For agents that have either already left the scene or have not yet appeared, their state is considered unobserved. Consequently, for a given vehicle, its corresponding node feature $v_{i}$ will either be $\left(x_{i},y_{i}\right)$ or the unobserved state (0,0).

The GCN structure is chosen because it excels at capturing spatial relationships and interactions between entities in a graph, making it ideal for modeling the complex interactions between multiple road agents in autonomous driving scenarios. After all the data are transformed into graphs, the GCN [37] is used to carry out the feature extraction operation. The layer-wise propagation rule for the GCN is given by the following equation:

(5) $$H^{\left(l+1\right)}=\sigma \left({\tilde{D}}^{-\frac{1}{2}}\tilde{A}{\tilde{D}}^{-\frac{1}{2}}H^{\left(l\right)}W^{\left(l\right)}\right)$$

where $H^{\left(l\right)}$ represents the activation matrix at the *l*-th layer, $H^{\left(l+1\right)}$ is the activation matrix at the (l+1)-th layer, $\tilde{A}$ is the adjacency matrix of the graph with added self-connections, $\tilde{D}$ is the degree matrix of $\tilde{A}$, $W^{\left(l\right)}$ is the weight matrix for the *l*-th layer, and σ denotes the activation function, such as the ReLU function. This rule is applied iteratively to propagate signals through the network.

## 7. Experiment

### 7.1. Training Details

One Nvidia GeForce RTX 3080 Ti GPU with 16GB memory is used in the work. The whole dataset is split into four different subsets according to the four map types, for each subset, and is trained for 50 epochs to generate the best result. We trained four independent models using these four recordings and tested each them on all four scenarios to evaluate the in-distribution and out-of-distribution performance of these models. Additionally, the models’ performances were compared at different times, i.e., morning, noon and afternoon. To deal with the data imbalance problem and maintain the same data distribution as the original dataset, stratified sampling was decided to launch on those four roundabout types, (each accounting for 25%) to obtain two new datasets and compare the performances of the trained models on the two new datasets. Additionally, we define our problem based on a 1s observation and 1s prediction, i.e., 25 frames for each.

In terms of the hyperparameters, we use a learning rate of 1 × 10^−3^, batch size of 256, and choose Adam as the optimizer with weight decay at 1 × 10^−5^. Apart from that, the input vectors consist of ten features, i.e., xCenter, yCenter, xVelocity, yVelocity, xAcceleration, yAcceleration, lonVelocity, latVelocity, lonAcceleration, and latAcceleration. To extract meaningful features from these high-dimensional data, a reduction in their dimensionality is required. In this study, Principal Component Analysis (PCA) [38] is employed to use two attributes instead of the whole ten inputs to represent the majority of road agent features in terms of motion forecasting because the inter-relationships between them were found, according to Figure 6.

During the GCN feature extraction process, combining and calculating the graph features for each agent into the MLP layer can be computationally intensive. To simplify this process, a modification is made: after applying the GCN, the information from all other agents is ignored, and we focus only on the target agent’s features. Specifically, only the $i_{target}^{th}$ dimension of the hidden layer values is selected and concatenated, where $i_{target}^{th}$ corresponds to the index of the target agent.

### 7.2. Input Format

The selected inputs are from four independent recordings with the tensor format B∗T∗2, with B=256 representing for the batch size and T=25 standing for 1 s observed data, and two-dimensional coordinates (x,y). The output’s size is the same as the input. When preparing for the dataset, agents are first chosen according to their types, i.e., excluding the VRUs (bicycle and pedestrian). As discussed before in Section 5.1, the VRUs account for very small percentage of the whole data. To select an agent *i* over the range $\left[t_{0},t_{n}\right]$, a sliding window approach is applied: the first 25 frames are chosen as the training set, and the following 25 frames are chosen as the testing set, where $t_{0}$ and $t_{n}$ represent the first frame and the last frame of agent *i*, respectively.

### 7.3. Coordinate Transform

Most methods use world spatial coordinates as the input of their models, but there exist some disadvantages to this straightforward method [39]. For example, world coordinates are absolute and can be complex to work with, especially in dynamic environments where many objects are moving simultaneously. Moreover, small errors in world coordinates can lead to significant inaccuracies in trajectory predictions, especially over long distances or durations. In this paper, the local coordinate system is used to transform our dataset. Firstly, each agent’s coordinates are calculated with respect to the target agent *a* at timestamp *k* in the training sequence for both training and predicting, including the target agent itself.

(6) $$P_{i,t_{j}}^{\prime }=\left\{\begin{array}{cc} \left(0,0\right) & \mathrm{if}\;i=a\;\mathrm{and}\;t_{j}=k\\ \left(x_{i,t_{j}}-x_{a,k},y_{i,t_{j}}-y_{a,k}\right) & \mathrm{otherwise} \end{array}\right.$$

where *i* is in the range of [1,n] representing different agents, *n* is the total number of agents. $t_{j}$ is a certain time step in the existing duration of agent *i*, and $x_{i,t_{j}}$ and $y_{i,t_{j}}$ represent the two-dimensional coordinates of agent *i* at $t_{j}$ moment. In this way, the coordinates of all agents are normalized in relation to the target agent at the beginning of training.

**Figure 12:**
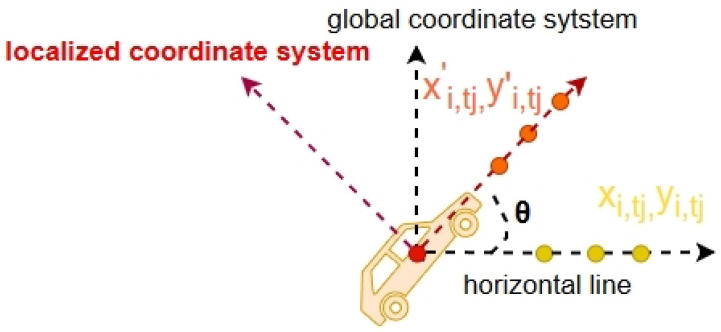
Illustration of the transformation from a global coordinate system to a localized coordinate system.

Subsequently, it has been ascertained that varying scenarios and road conditions exert a considerable influence on the alterations in trajectory prediction, encompassing both longitudinal and lateral dimensions. Consequently, there arises a necessity to establish a novel target-based coordinate system that registers the forward momentum of the target vehicle as the principal axis of variance. By adopting this approach, one can efficaciously transform the global coordinate framework into a localized one.

(7) $$\begin{array}{cc} x_{i,t_{j}}^{\prime } & =x_{i,t_{j}}\cdot \cos\left(\theta \right)-y_{i,t_{j}}\cdot \sin\left(\theta \right)\\ y_{i,t_{j}}^{\prime } & =x_{i,t_{j}}\cdot \sin\left(\theta \right)+y_{i,t_{j}}\cdot \cos\left(\theta \right), \end{array}$$

where θ is the angle between the agent’s driving direction and the x-axis (i.e., horizontal line) of the global coordinate (shown in Figure 12).

The final step is to complete normalization. After the following transformation, trajectory data are obtained, whose scale is relatively small (the difference of all data in one training sequence is close to the level of 1 × 10^−2^), which is not beneficial for training the network. So, the normalization of the whole data is required in the range [0,1] using a MIN-MAX scaler.

(8) $$\begin{array}{cc} x_{i,t_{j}}^{\prime } & =\frac{x_{i,t_{j}}-\mu_{x}}{\sigma_{x}}\\ y_{i,t_{j}}^{\prime } & =\frac{y_{i,t_{j}}-\mu_{y}}{\sigma_{y}} \end{array}$$

where $\mu_{x}$ and $\sigma_{x}$ are the mean and variance of transformed x coordinates in the whole dataset, respectively, as well as y coordinates.

### 7.4. Metrics and Loss Function

For this trajectory prediction problem, the following standard metrics are used in our work.

1. ADE (Average Displacement Error): The average Euclidean distance between the predicted trajectory and the real trajectory.
  $$ADE=\sqrt{\frac{\sum_{k=1}^{r}\sum_{i}^{n}{\left(D_{i}^{dist}\right)}^{\left(k\right)}}{n}}$$
  where n indicates the number of vehicles, r indicates the prediction step, and $D_{i}^{dist}$ indicates the Euclidean distance between the actual and predicted coordinates of vehicle i.
2. FDE (Final Displacement Error): Euclidean distance between trajectory prediction endpoint and true value.
  $$FDE=\sqrt{{\left(x^{pred}-x^{truth}\right)}^{2}+{\left(y^{pred}-y^{truth}\right)}^{2}}$$
  where $\left(x^{pred},y^{pred}\right)$ is the end point of the predicted trajectory, and $\left(x^{truth},y^{truth}\right)$ is the end point of the actual trajectory.

In this problem, the basic MSE (Mean Squared Error) is used as the main loss function.

$$L=\frac{\sum_{i=1}^{n}{\left(x_{i}^{pred}-x_{i}^{truth}\right)}^{2}+{\left(y_{i}^{pred}-y_{i}^{truth}\right)}^{2}}{n}$$

where n is the total number of data size, and $\left(x_{i}^{pred},y_{i}^{pred}\right)$ and $\left(x_{i}^{truth},y_{i}^{truth}\right)$ are defined as above.

## 8. Results and Discussion

The following four Table 4, Table 5, Table 6 and Table 7 present the results of our four separate models trained on a specific domain (roundabout types 0, 1, 2 and 9) and tested on both in-distribution and out-of-distribution domains across four test sets. The tables compare the performance of three models—GCN, LSTM, and our one—using ADE and FDE metrics. The goal of this comparison is to evaluate the generalization capabilities of each model when trained on one domain and tested on others, thereby assessing their robustness in different roundabout environments.

### 8.1. Advantages of Our Model

According to Table 4, Table 5, Table 6 and Table 7, our model demonstrates a remarkable improvement over the baseline GCN and LSTM models under the evaluation of ADE and FDE.

- Accuracy: Our model consistently yields a lower ADE and FDE across all recording types compared to both GCN and LSTM. While GCN exhibits significantly higher error rates, reflecting its limited effectiveness in capturing the detailed dependencies necessary for accurate trajectory prediction, LSTM performs better but still falls short, especially in complex scenarios. By achieving roughly 50% lower errors than GCN and 20% lower than LSTM, our model is proved to be better than these two baseline models in trajectory accuracy.
- Robustness and Stability: The performance of GCN is particularly worse mainly because of its rigid architecture and limitations in sequential learning, which are less adaptable to complexities, especially with a large number of agents in trajectory data. Although LSTM performs better due to its sequential approach, it still obtains a higher ADE and FDE in more complex and variable scenarios (such as recording 2 and 9) because it does not have the ability to consider the effect from neighboring agents. In contrast, our model maintains stable and lower error rates across all recordings, indicating greater robustness and the ability to handle diverse and complex patterns effectively.
- Application Suitability: In future applications, where prediction reliability is crucial, our model offers a clear advantage. It outperforms both GCN and LSTM, and combines both advantages in delivering lower error rates consistently, making it highly suitable for scenarios requiring dependable, real-time predictions.

### 8.2. Domain Generalization Analysis

In this part, the domain generalization problem is analyzed primarily in terms of the model. By observing these tables, several general trends and patterns emerge regarding the performance of the model trained and tested on different scenes. Firstly, it is noticed that the lowest ADE and FDE values are generally observed when the training and testing scenes coincide. This indicates that the models perform best when applied to the same type of environment they were trained on, highlighting the importance of scene-specific training data. For instance, the model trained and tested on roundabout type 0 shows relatively low ADE and FDE values (0.81 and 1.06, respectively), suggesting high accuracy in predictions for that specific environment. Similarly, the model trained and tested on roundabout type 9 is made up of simple traffic patterns with a high presence of cars, giving rise to low error values (0.16 ADE and 0.31 FDE).

The models trained on complex scenes like roundabout type 1 have higher error values when tested with other roundabout types. The main reason for this is that the sophisticated traffic dynamics and multiple agent types in roundabout type 1 make it difficult to generalize over other environments. A model trained on roundabout type 2 would have an ADE of 0.28 and FDE of 0.49 when tested in itself. In roundabout type 9, it can be observed that these two metrics are much smaller than that in roundabout type 0 and 1, which indicates some shared characteristics that facilitate better performance. Meanwhile, the prediction for roundabout type 2 based on the model trained on roundabout type 1, which exhibits much more high frequencies of acceleration and deceleration than other roundabout types, is surprisingly not good, indicated by the highest error values when tested in all models.

Overall, the results in these four tables show that the four models generally exhibit lower prediction errors when applied to roundabout types with similar traffic dynamics and agent distributions as their training data. This underscores the importance of accounting for specific scene characteristics during model training to enhance predictive accuracy and generalizability. Additionally, predictable environments with homogeneous agent types, such as roundabout type 9, tend to yield more accurate predictions across the four independent models, further emphasizing the benefit of training on stable and representative data.

### 8.3. Visualization Analysis

**Figure 13:**
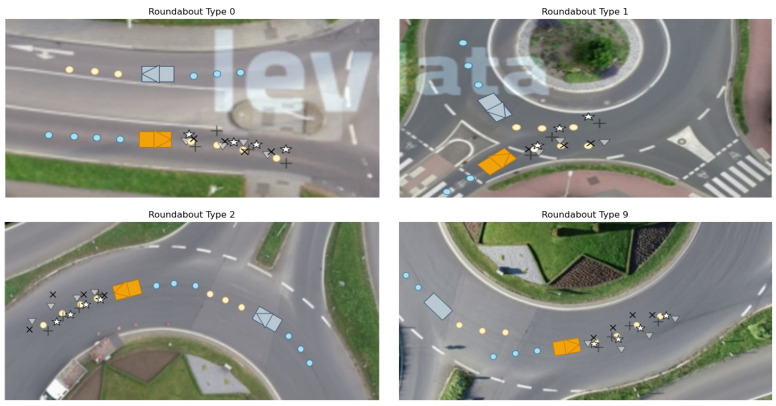
Visualized Prediction Results: (target, neighboring agents). … and … are the trajectory history and labels of all road agents. ▽ are the predicted trajectories of the targets in model 0. × represents model 1, and + and ★ represent model 2 and model 9, respectively.

In Figure 13, for roundabout type 0, the predictions by the four independent models are clustered closely around the actual trajectory, as indicated by the yellow dots. The model trained on roundabout type 0 might be displaying the highest accuracy, given its familiarity with the scene’s dynamics. The model trained on roundabout type 9 appears to predict the trajectory accurately, which might indicate that the traffic behavior in roundabout types 0 and 9 shares similarities that the model can generalize. The divergence between predictions and the ground truth is minimal, suggesting that the models can capture the necessary patterns in such simple scenarios.

For roundabout type 1, the trajectories predicted in this scene show greater divergence, which could be due to the complexity of the roundabout type itself, such as more lanes or higher traffic density. The model trained on roundabout type 1 likely demonstrates better performance compared to the others, as it would be specialized to the specific complexities of the roundabout type. Models trained on other roundabout types (0, 2, and 9) might struggle to adapt their predictions to the unique traffic patterns of roundabout type 1, hence the greater variance in predicted trajectories.

For roundabout type 2, it presents a roundabout with a central island. The complexity of roundabout interactions is reflected in the more scattered predictions. The models trained on this roundabout type and similar type 9 are expected to have learned the nuances of their traffic flow, potentially resulting in closer predictions to the ground truth. Other models (0 and 1) seem to have divergent predictions, indicating a lower ability to capture the specific traffic behaviors of this scene, which may be quite different from those in their training data.

For roundabout type 9, the predictions in this scene are relatively close to each other, suggesting that all the models find this roundabout type easier to predict or that there is some commonality in the traffic dynamics that all models are able to grasp. Given that the model trained on roundabout type 9 shows predictions closely following the ground truth, it suggests high accuracy and an understanding of this scene’s particular characteristics. Other models, even though trained on different scenes, seem to provide reasonable predictions for this recording, which may indicate that the traffic patterns in roundabout type 9 are less unique or that the behaviors here are well represented in the training datasets of the other models.

## 9. Conclusions

In this paper, we conducted an analysis of the rounD dataset to enhance our understanding of trajectory prediction for autonomous vehicles in complex roundabout environments. By implementing a trajectory prediction framework that combines GRU networks with graph-based modules to capture vehicle interactions, the generalizability of a model was assessed across diverse training and testing datasets. Our extensive experiments demonstrated that varying data distributions—including differences in road configurations and recording times—significantly influence a model’s prediction accuracy and robustness. It can be found that models trained on a broader range of data exhibited stronger generalization to unseen scenarios, emphasizing the necessity for diverse and representative training datasets for effective domain generalization in autonomous driving.

### 9.1. Limitations

While our study provides valuable insights, there are several limitations to consider. Although two additional backbone models were included to enhance our evaluation, the reliance on the rounD dataset alone may still limit the generalizability of our findings. The dataset may not fully capture all real-world driving conditions, particularly those influenced by extreme weather events, varying traffic densities, or less common road configurations. Furthermore, the current study does not address potential issues of data sparsity in certain scenarios, which could affect model performance and robustness.

### 9.2. Future Work

To address these limitations, future research should focus on expanding the dataset to encompass a wider variety of driving conditions, including scenarios with diverse environmental factors such as inclement weather and night-time driving. Furthermore, the aim is to contribute to the development of domain generalization techniques by exploring advanced methods that enhance the model’s ability to generalize across different environments and conditions. This includes investigating the integration of multi-modal data, such as sensor fusion from LiDAR and camera inputs, to bolster the robustness of trajectory prediction models. Additionally, exploring advanced techniques for continual learning and domain adaptation may provide pathways to improve model performance in rapidly changing environments. Through these efforts, the field of trajectory prediction in autonomous vehicles is aimed to be advanced, fostering models that are not only accurate but also resilient across dynamic and unpredictable scenarios.

## Figures and Tables

**Table 1:** This table contains metadata for each recording. The metadata provide a general overview, e.g., of the time of recording, the road section considered, and the total number of objects tracked.

Name	Description	Unit
recordingId	Unique identifier for the recording.	[-]
locationId	Unique identifier for the recording location.	[-]
frameRate	Video frame rate used during recording.	[hz]
speedLimit	Speed limit of the lanes in the recording.	[m/s]
weekday	Day of the week when the recording occurred.	[-]
startTime	Starting hour of the recording.	[hh]
duration	Total time of the recording.	[s]
numTracks	Number of tracked objects.	[-]
numVehicles	Number of tracked vehicles (cars, trucks, vans, trailers).	[-]
numVRUs	Number of tracked vulnerable road users (pedestrians, bicycles, motorcycles).	[-]
latLocation	Approximate latitude of the recording.	[deg]
lonLocation	Approximate longitude of the recording.	[deg]
xUtmOrigin	UTM X coordinate origin for the recording location.	[m]
yUtmOrigin	UTM Y coordinate origin for the recording location.	[m]
orthoPxToMeter	Conversion factor from ortho pixels to UTM meters.	[m/px]

**Table 2:** This table contains all the information of each agent, such as current position, velocity and acceleration.

Name	Description	Unit
recordingId	Unique recording identifier.	[-]
trackId	Unique track identifier for each recording.	[-]
frame	Frame number for the data.	[-]
trackLifetime	Age of the track at the current frame.	[-]
xCenter	X coordinate of the object’s centroid.	[m]
yCenter	Y coordinate of the object’s centroid.	[m]
heading	Object’s heading angle.	[deg]
width	Object’s width.	[m]
length	Object’s length.	[m]
xVelocity	Velocity along the x-axis.	[m/s]
yVelocity	Velocity along the y-axis.	[m/s]
xAcceleration	Acceleration along the x-axis.	[m/s^2^]
yAcceleration	Acceleration along the y-axis.	[m/s^2^]
lonVelocity	Longitudinal velocity.	[m/s]
latVelocity	Lateral velocity.	[m/s]
lonAcceleration	Longitudinal acceleration.	[m/s^2^]
latAcceleration	Lateral acceleration.	[m/s^2^]
initialFrame	Start frame of the track.	[-]
finalFrame	End frame of the track.	[-]
numFrames	Total lifetime of the track in frames.	[-]
class	Object class (e.g., Car, Pedestrian, Bicycle).	[-]

**Table 3:** Data volume for different roundabout types.

Roundabout Type	Index	Number of Records (Million)
0	0	0.3
1	1	0.1
2	2–8	0.2, 0.3, 0.2, 0.2, 0.3, 0.3, 0.2
9	9–23	0.3, 0.3, 0.2, 0.2, 0.2, 0.3, 0.9, 0.1, 0.3, 0.3, 0.2, 0.3, 0.3, 0.2, 0.2

**Table 4:** Result comparison of models trained on one particular domain (i.e., recording type 0) and tested on both in-distribution and out-distribution domains.

ADE\FDE	Roundabout Type 0			
Train	0			
Test	0	1	2	9
GCN	2.33\5.42	2.87\4.28	3.21\5.44	3.02\5.32
LSTM	0.95\1.12	1.05\1.33	1.12\1.27	1.15\1.58
Ours	**0.81\1.06**	**0.88\1.14**	**0.99\1.05**	**1.09\1.34**

**Table 5:** Result comparison of models trained on one particular domain (i.e.,Recording Type 1) and tested on both in-distribution and out-distribution domains.

ADE\FDE	Roundabout Type 1			
Train	1			
Test	0	1	2	9
GCN	3.02\3.82	2.45\4.19	3.67\5.67	4.28\5.12
LSTM	1.12\1.38	0.91\1.29	1.44\1.76	1.52\2.02
Ours	**1.08\1.24**	**0.87\1.34**	**1.29\1.96**	**1.46\1.78**

**Table 6:** Result comparison of models trained on one particular domain (i.e., recording type 2) and tested on both in-distribution and out-distribution domains.

ADE\FDE	Roundabout Type 2			
Train	2			
Test	0	1	2	9
GCN	4.35\5.18	3.53\4.44	2.89\3.69	3.17\4.21
LSTM	1.35\1.73	1.45\1.39	0.34\0.77	0.95\0.97
Ours	**1.34\1.69**	**1.23\1.43**	**0.28\0.49**	**0.79\0.82**

**Table 7:** Result comparison of models trained on one particular domain (i.e., recording type 9) and tested on both in-distribution and out-distribution domains.

ADE\FDE	Roundabout Type 9			
Train	9			
Test	0	1	2	9
GCN	3.57\4.01	2.99\4.05	2.91\4.06	2.53\1.99
LSTM	1.34\1.68	1.22\1.45	0.95\1.39	0.63\0.41
Ours	**1.12\1.42**	**0.93\1.43**	**0.57\0.62**	**0.16\0.31**

## Data Availability

Data are contained within this article.
